# Diversity of marine bacteria producing beta-glucosidase inhibitors

**DOI:** 10.1186/1475-2859-12-35

**Published:** 2013-04-17

**Authors:** Sony Pandey, Ayinampudi Sree, Soumya Suchismita Dash, Dipti Priya Sethi, Lipsa Chowdhury

**Affiliations:** 1 Environment and Sustainability Department, CSIR - Institute of Minerals and Materials Technology, Bhubaneswar, Odisha, 751013, India

**Keywords:** Glucosidase inhibitor, Diversity, Microbial extracts, Phyla, Marine microorganisms, Anti-diabetics, Anti-obesity, Marine sponge, *Aka coralliphaga*, *Sarcotragus fasciculatus*

## Abstract

**Background:**

Beta-glucosidase inhibitors are being extensively studied for use as anti-diabetics, anti-obesity and anti-tumour compounds. So far, these compounds have been reported in large numbers from plants, mushrooms, algae and fungi. There are very few reports of such inhibitors from bacteria in the open literature, particularly marine bacteria; although the best known inhibitor deoxynojirimycin was isolated from bacilli and actinomycete. Through this study, we tried to discover the diversity of microbial associates of marine sponge and sediment producing β-glucosidase inhibitors.

**Results:**

We found 41 (22.7%) out of 181 bacteria, produced such inhibitors. The inhibitors are abundant in bacterial associates of marine sponge *Aka coralliphaga*. When these bacteria were phylogenetically analyzed, it was found that marine bacteria producing glucosidase inhibitors belong to the phylum Firmicutes (23), Actinobacteria (9), Proteobacteria (7) and Bacteroidetes (1).

**Conclusion:**

A significant number of marine bacteria belonging to a wide range of bacterial taxa were found to produce β-glucosidase inhibitors. These compounds are abundantly present in bacteria of the phylum Firmicutes followed by the phylum Actinobacteria. The results nurture a hope of finding new compounds, which can inhibit glucosidases, in the bacterial domain of marine organisms. Thus, marine microbial cells can be utilized as producers of pharmacologically essential enzyme inhibitors.

## Background

Glucosidase inhibitors are emerging as important therapeutic agents since they interfere with crucial metabolic functions in biological systems, and have widespread applications in pharmacology and agriculture. They also provide useful insights into the role of glucosidase in living systems and enzyme action mechanism. Their therapeutic applications alone range from the treatment of infections, to metabolic and genetic disorders like – viral infections of HIV/Influenza, metastatic cancer, diabetes, obesity and lysosomal storage diseases [1]. At present nojirimycin and its derivatives are being extensively used as β-glucosidase inhibitors. Some species of *Streptomyces* and *Bacillus* produce antimicrobial compounds like validamycin and nojirimycin, which showed β-glucosidase inhibition later on [2-4]. Yet the search for β-glucosidase inhibitors in microorganisms is very much limited, though natural sources like plant extracts, microalgae, cyanobacteria and mushrooms have been explored [5-8].

The natural compounds exhibiting β-glucosidase inhibition belong to a variety of chemical classes like disaccharides, iminosugars, carbasugars, thiosugars and non-sugar derivatives [9]. There are naturally occurring sugar mimics in the plant and microbial world which have the potential to act as β-glucosidase inhibitors, since they are structural analogues of natural sugar substrates [10]. Beta-glucosidase inhibitors exhibit a range of structure and functions in nature, so it is intriguing to search for these inhibitors in natural resources, which have the potential to produce different structures. Marine microflora is one such natural resource which remains to be explored for the presence of β-glucosidase inhibitors. Microbes living in the marine environment survive under extreme conditions of temperature, pressure and nutritional competition; and hence they possess metabolic capabilities, which may be different from their terrestrial counterparts. The inhibitors of β-glucosidases are expected to be diverse in the marine environment since this enzyme is widespread and diverse [11]. In the past few decades, marine microbes from the sponges and sediment have given several novel therapeutic molecules [12]. However, except for a report by Imada and Okami 1995 [13], on a deep-sea actinomycete isolate producing β-glucosidase inhibitor, we did not find any literature in this area. This prompted us to seek β-glucosidase inhibitors in marine sponge and sediment associated bacteria.

When we investigated the microbial associates of sponges and sediments using our new method of β-glucosidase inhibition assay, we found β-glucosidase inhibitors in several marine microbial extracts [14]. Many authors have emphasized that phylogenetic diversity is the source of varied biological activity [15-17]. Thus, the objective of this study was to find the taxonomic groups of bacteria, isolated from marine sponges and sediment samples, involved in the inhibition activity. To the best of our knowledge, this report is the first to point out the phylogenetic diversity of marine microbes producing β-glucosidase inhibitors.

## Results and discussion

Glucosidases catalyze the cleavage of glycosidic bonds involving α- and β-linked glucose units or the bonds between sugars and a non-carbohydrate aglycone moiety. Beta-glucosidases play a crucial role in several biochemical processes like degradation of polysaccharides, lysosomal glycoconjugate catabolism, glycoprotein and glycolipid processing. Glucosidase inhibitors have become the subject of intense scrutiny since the isolation of deoxynojirimycin in 1966, because of their profound effect on glycoprotein processing, oligosaccharide metabolism, cell-cell and cell-virus recognition processes [18,19]. Our aim was to find the diversity of marine bacteria producing β-glucosidase inhibitors in response to the presence of this enzyme in their environment, and we indeed found highly diverse microbial population possessing the ability to produce these enzyme inhibitors.

Out of the 181 isolates tested 41 (22.7%) organisms showed β-glucosidase inhibition potential; of these 41 isolates, 27 belonged to sediment samples, 6 and 8 respectively from the sponge *Sarcotragus fasciculatus* and *Aka coralliphaga* of Bay of Bengal. Table 1 presents quantitative data on the number of samples screened, beta-glucosidase inhibiting organisms and their diversity at phyla level. Our results show that 22.7% of the microorganisms screened are able to inhibit β-glucosidase, which further strengthens the belief that glucosidase inhibitors are widespread in the plant and microbial world [10]. Twenty seven (24.5%) out of 110 isolates from sediment samples were positive for beta-glucosidase, 6 out of the 41 (14.6%) isolates from the sponge *Sarcotragus fasciculatus* and 8 out of 30 (26.6%) from the sponge *Aka coralliphaga* produced the inhibitors. Since long, the marine sponge associated microbes have been projected as a prolific source of bioactive compounds [11]; our study shows that the sediment is also a good source of the inhibitor compound.

**Table 1:** Data on screening results

**Sample**	**No. of isolates screened**	**No. of isolates positive**	No. of different species obtained*	**No. of genus obtained**	**No. of different phyla obtained**
SD1 Sediment	27	11	7 (2)	9	Firmicutes – 3
					Actinobacteria – 4
					Proteobacteria – 3
					Bacteroidetes - 1
SD2 Sediment	30	13	9 (2)	5	Firmicutes – 10
					Actinobacteria – 2
					Proteobacteria – 1
GD Sediment	53	3	2 (1)	3	Firmicutes – 1
					Actinobacteria – 1
					Proteobacteria – 1
Sponge *Sarcotragus fasciculatus*	41	6	3 (1)	3	Firmicutes – 5
					Proteobacteria – 1
Sponge *Aka coralliphaga*	30	8	7 (1)	5	Firmicutes – 5
					Actinobacteria – 2
					Proteobacteria – 1
Total	181	41	26^^^	19^^^	

^*^Number in brackets are unidentified at species level.

^^^*The numbers exclude overlapping genus and species obtained in different samples.*

**Figure 1:**
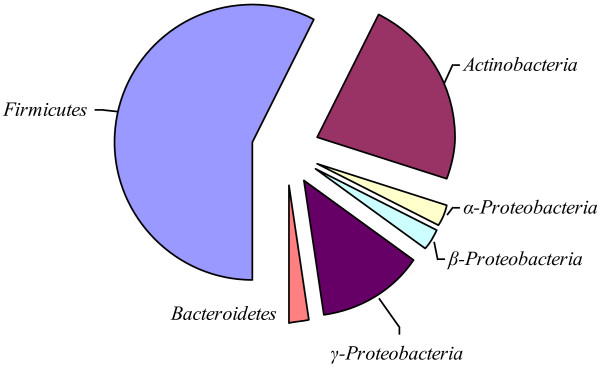
**Distribution of β-glucosidase inhibiting marine isolates in different bacterial phyla showing Firmicutes making up the major fraction followed by Actinobacteria, Proteobacteria and Bacteroidetes.** Within Proteobacteria, the γ-Proteobacteria constitutes the major fraction.

**Figure 2:**
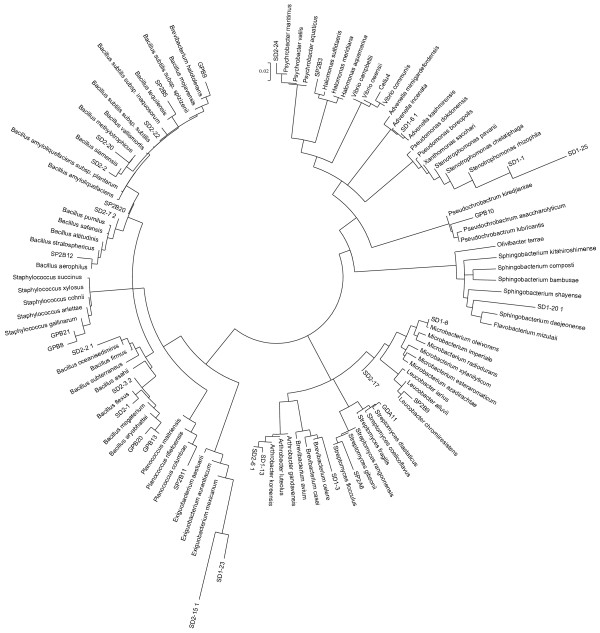
**Phylogenetic tree involving all the taxa showing beta-glucosidase inhibition.** The analysis involved 117 nucleotide sequences. All ambiguous positions were removed for each sequence pair after performing pairwise deletion. The evolutionary history was inferred using the Neighbor-Joining method [20]. Bootstrap test was performed on the clusters in 1000 replicates [21]. The evolutionary distances were computed using the p-distance method [22] and are in the units of the number of base differences per site. The optimal tree with the sum of branch length = 2.39 is shown. The tree was drawn to scale in circular branch style, with branch lengths in the same units as those of the evolutionary distances used to infer the phylogenetic tree. There were a total of 1580 positions in the final dataset.

All the active isolates were identified by partial 16S rDNA sequencing in the V1-V3 variable region and confirmed by culture characterization (data not shown). While Table 1 shows the quantitative data of the screening activity, Table 2 shows the qualitative data on the activity and identity of the positive isolates. The distribution of active isolates among different bacterial phyla is depicted in Figure 1. Based on the 16S rDNA sequence data, a phylogenetic tree was drawn (Figure 2).

**Table 2:** β-Glucosidase inhibition activity and identity of the marine isolates

**Sl. No.**	**Strain**	β-Glucosidase inhibition (in mm*)	**Identity (%)**	**Closest match**	**GenBank accession number**
1	SD1-1	4	99.8	***Stenotrophomonas rhizophila***	JQ359448
2	SD1-3	4	100	***Arthrobacter koreensis***	JQ409500
3	SD1-6(1)	5	99.0	***Advenella kashmirensis***	JQ359449
4	SD1-8	5	99.7	***Microbacterium oleivorans***	JQ359450
5	SD1-13	4	100	***Arthrobacter koreensis***	JQ409501
6	SD1-14(1)	6	99.8	***Planomicrobium okeanokoites***	JQ409502
7	SD1-17	4	97.3	***Dietzia maris***	JQ409503
8	SD1-18	4	96.0	***Chryseomicrobium sp.***	JQ409504
9	SD1-20(1)	4	90.0	***Sphingobacterium sp.***	JQ409505
10	SD1-23	4	97.3	***Exiguobacterium marinum***	JQ409506
11	SD1-25	4	98.5	***Stenotrophomonas rhizophila***	JQ409507
12	SD2-1	4	100	***Bacillus flexus***	JQ409508
13	SD2-2(1)	5	99.5	***Bacillus oceanisediminis***	JQ409509
14	SD2-2(2)	5	100	***Bacillus siamensis***	JQ409510
15	SD2-3(2)	6	100	***Bacillus flexus***	JQ409511
16	SD2-5	4	100	***Bacillus sp.***	--
17	SD2-6(1)	5	100	***Arthrobacter koreensis***	JQ409512
18	SD2-7(2)	5	100	***Bacillus stratosphericus***	JQ409513
19	SD2-15(1)	4	91.0	***Exiguobacterium sp.***	JQ409514
20	SD2-17	4	99.1	***Bacillus oceanisediminis***	JQ409515
21	SD2-18	4	99.8	***Microbacterium esteraromaticum***	JQ409516
22	SD2-20	4	98.7	***Bacillus methylotrophicus***	JQ409517
23	SD2-22	5	99.8	***Bacillus subtilis subsp. inaquosorum***	JQ409518
24	SD2-24	4	99.7	***Psychrobacter maritimus***	JQ409519
25	GDN4	5	99.6	***Vibrio communis***	JQ409520
26	GDB16	5	95	***Bacillus sp.***	JQ409521
27	GDA11	4	99.6	***Streptomyces coelicoflavus***	JQ409522
28	GPB8	4	99.7	***Staphylococcus gallinarum***	JQ409523
29	GPB9	4	99.7	***Bacillus subtilis subsp. spizizenii***	JQ409524
30	GPB10	4	96.0	***Pseudochrobactrum sp.***	JQ409525
31	GPB13	4	100	***Bacillus aryabhattai***	JQ409526
32	GPB20	4	99.6	***Bacillus aryabhattai***	JQ409527
33	GPB21	4	100	***Staphylococcus gallinarum***	JQ409528
34	SP2B3	6	97.8	***Halomonas sulfidaeris***	JQ409529
35	SP2B5	4	99.4	***Bacillus tequilensis***	JQ409530
36	SP2B6	6	95.5	***Bacillus sp.***	--
37	SP2B9	6	99.7	***Leucobacter chromiiresistens***	JQ409531
38	SP2B11	5	98.8	***Planococcus rifitoensis***	JQ409532
39	SP2B12	5	99.5	***Bacillus stratosphericus***	JQ409533
40	SP2B20	6	100	***Bacillus amyloliquefaciens subsp. amyloliquefaciens***	JQ409534
41	SP2A6	4	97.6	***Streptomyces rangoonensis***	JQ409535

* Standard deviation ± 0.5 mm.

An explanation to the observation of a wide range of microbial taxa involved in this activity could lie in the competitiveness of the ecosystem. In the ocean ecosystem, decomposable organic material is scarcely available so most of the heterotrophic bacteria in the ecosystem would secrete the glucosidase enzyme; and in order to compete with others for the small amount of nutrition, they would also secrete a variety of glucosidase inhibitors. The role of organic carbon availability is evident in the study by Liu et *al.* 2008, where the β-glucosidase activity of mangrove sediments was compared with other water bodies including deep sea. The activity was found to be highest in the mangrove ecosystem due to the abundant availability of decomposable organic matter [23].

An interesting observation from this study is that very few Actinomycetes (21.9%), contrary to their popular characteristic of bioactivity, have shown glucosidase inhibition as compared to Firmicutes bacteria (58.5%) [15,16]. Observation of Table 2 shows glucosidase inhibition by species belonging to diverse genera; however only two species of *Streptomyces* were active. Six genera of Firmicutes bacteria – *Planomicrobium, Chryseomicrobium, Exiguobacterium, Bacillus, Staphylococcus* and *Planococcus*; and five genera of Actinobacteria – *Leucobacter, Streptomyces, Microbacterium, Arthrobacter and Dietzia*, exhibited β-glucosidase inhibition. The bioactive isolates from sediment samples belonged to four phyla, whereas only three phyla were detected in the sponge samples (Table 1).

According to Stein et *al.* 1984, deoxynojirimycin has been isolated earlier from several species of *Bacillus* (phylum Firmicutes) and *Streptomyces* (phylum Actinobacteria) [4]. Even in our study majority of the active isolates belong to these phyla but, we report number of genera from these phyla to produce such natural compounds. Our finding is consistent with the general perception that, Firmicutes and Actinobacteria are better competitors in the natural environment since they are prolific producers of antibiotics and enzyme inhibitors.

A significant number of isolates - 7 out of 41 (17.1%), belong to phylum Proteobacteria with representatives of all three classes α-, β- and γ-Proteobacteria. Species of *Bacillus* and *Streptomyces* are already known for their ability to produce diverse bioactive compounds; our finding is more important in the context of genera like *Staphylococcus, Stenotrophomonas, Pseudochrobactrum, Advenella, Dietzia* and *Chryseomicrobium,* which are hitherto unknown as producers of antimicrobial and anti-enzyme compounds. In fact, our study is the first to report glucosidase inhibition in many genera of bacteria, which have so far shown enzyme, cytotoxic, antimicrobial, antioxidant, biosurfactant and metal resistance properties only. Like, marine isolates of *Planomicrobium, Planococcus, Microbacterium, Arthrobacter, Halomonas and Psychrobacter* have shown weak cytotoxic activity earlier [24]. Enzymes like chitinase, xylanase, keratinase have been reported from *Planococcus rifitoensis*[25], *Arthrobacter sp.*[26] and *Microbacterium sp.*[27] respectively. Interestingly, *Sphingobacterium* has previously shown thioglucosidase activity [28] and recently α-glucosidase has also been reported from a strain of *Halomonas*[29]. In fact, many *Halomonas* species are reported in literature with varied activity, for example, a marine derived *Halomonas* strain produced certain antimicrobial and cytotoxic compounds with the addition of anthranilic acid in the fermentation medium [30]. Our study is also the first to report very strong glucosidase inhibition activity in a chromium resistant species of *Leucobacter* from the sponge *Aka coralliphaga*. A number of other members from this genus have shown to have chromium tolerance and recently a species of *Leucobacter* has been reported to have potential biosurfactant activity [31,32].

Natural products from microbial associates of marine sponges and sediments are attractive because of the metabolic diversity they exhibit and scalability of the production process using biotechnological methods. Hence, the supply problem associated with marine invertebrates and plankton can be overcome [33]. Although the role of glucosidase inhibitors in marine sponge is not clear and further work is needed, we speculate that these extracellular inhibitors are released to prevent the other microbes from utilizing the carbohydrate carbon. We believe our revelations form only the tip of an iceberg, and there are many more cell factories hidden in the deep sea.

## Conclusion

This study throws light on the microbial taxa which are hitherto not known for producing such compounds. Thus, within the realm of possibility we may assume these microorganisms have a potential to produce new glucosidase inhibitor compounds. Glucosidases are central to carbohydrate metabolism and efficient utilization of carbon sources determines the viability of microbes in any environment. In order to survive in competitive environments like the marine ecosystem, the microbial cells need to produce inhibitors against the glucosidases of other microbes. Sparse information is available on the glucosidase inhibition activity of the diverse microbial phyla found in the depths of ocean for comparison of our results. Probably the missing link between the phylogenetic diversity and functional diversity can be established, if all these inhibitors are structurally identified and their reaction mechanisms established.

## Methods

### Bacterial strains and culture conditions

A total of 181 marine microorganisms isolated from marine sediment (110), sponge *Sarcotragus fasciculatus* (41) and sponge *Aka coralliphaga* (30) were selected for this study, out of a collection of marine cultures isolated from the Bay of Bengal on the east coast of India. All the organisms grew on Nutrient Agar (Hi Media, Mumbai) media prepared in 50% aged natural seawater at 30°C within 48–72 hrs.

### Extraction of metabolites

The marine cultures were grown in 50 ml Nutrient Broth prepared in 50% natural seawater and incubated at 30°C in 200 rpm shaker for 48 hrs. The cell biomass was centrifuged at 9000 rpm for 20 min, and the supernatant containing the secondary metabolites was collected in a 250 ml flask; followed by mixing 10% diaion HP-20 (Sigma) and magnetic stirring for 30 min. The contents of the flask were packed in a glass column, washed with 15 ml distilled water, and eluted with 20 ml methanol. The methanol fractions were evaporated in a rotary evaporator (Heidolph, Germany) and dissolved in DMSO, to make a final concentration of approx. 1 mg/ml and stored at - 20°C.

### Beta-glucosidase inhibition assay

The assay was performed as described by Pandey et *al.* 2013 [14]. Briefly, 10 ml enzyme agar solution was prepared with 0.7% agar powder dissolved at 80-100°C in 0.1 M sodium acetate buffer, followed by the addition of 0.06% FeCl_3_ and 0.01 U/ml enzyme β-glucosidase added at 60°C, and the solution was poured into petri plates. Then 5 μl of the samples were inoculated on the surface of the agar plate and incubated at room temperature for 15 min, followed by the addition of 0.2% esculin solution and again incubated at room temperature for 30 min for enzyme-substrate reaction. DMSO without extract was used as negative control, and the positive control was 0.75 μg conduritol β-epoxide. The results were recorded by measuring the zone size. These experiments were repeated thrice with some extracts to check the reproducibility of results.

### Microbial identification

All the cultures which showed a positive zone of inhibition were identified by morphological characterization and 16S rRNA gene sequencing. DNA was isolated from the pure cultures by the method described by Pitcher et *al.,* 1989 [34]. Purified genomic DNA was subjected to 16S rRNA gene amplification using the universal primers 27F and 1492R in a thermal Cycler (Eppendorf). The PCR amplification was performed in 25 μl reaction mixture containing 10 pmol of both the primers, 100–150 ng DNA, 5 mM dNTPs, 1X Taq polymerase buffer containing 15 mM MgCl_2_ and 0.5 μl of Taq DNA polymerase (5 U/μl, Fermentas). The PCR programme was as follows: initial denaturation at 94°C for 5 min, 30 cycles of 94°C for 1 min, 50°C for 1 min, 72°C for 2 min and final elongation at 72°C for 10 min. The PCR product was separated on 1% agarose gel and the DNA fragments were extracted using Gel Extraction kit (Qiagen) as per the manufacturer’s instructions. The purified 16S rDNA was sequenced at Merck Biosciences, Bangalore and CSIR-IMMT in Beckman Coulter CEQ 8000 genetic analysis system. The sequences thus obtained were aligned with the GenBank sequences using BLAST program in NCBI (http://blast.ncbi.nlm.nih.gov) and the EzTaxon server 2.1 (http://147.47.212.35:8080/) [35], to obtain the closest matching type strain sequences from the database. The identity of the strain was established based on the sequence identity and corresponding morphological characters.

### Phylogenetic analysis

The 16S rDNA sequences of the closest relatives of all the marine cultures showing positive glucosidase inhibition were retrieved from EzTaxon, and multiple sequence alignment was performed using Clustal_X program [36]. The evolutionary relatedness of all the strains was inferred using the Neighbor-Joining method, distance calculated using the p-distance method, followed by bootstrap test in 1000 replicates for each cluster and the phylogenetic tree was drawn in *MEGA5.0* program available at http://www.megasoftware.net/[37].

## Competing interests

The authors declare no competing interests.

## Authors’ contributions

SP contributed to the experimental design, data acquisition, troubleshooting, analysis and interpretation of data, as well as drafting the manuscript. AS conceived the work, collected samples, and advised on the manuscript. SSD, DPD and LC helped in sample preparations and execution of work. All the authors have read and approved the final manuscript.
